# Psychoanalytic core competence

**DOI:** 10.3389/fpsyg.2015.00356

**Published:** 2015-03-27

**Authors:** Karoline Parth, Henriette Loeffler-Stastka

**Affiliations:** Advanced Postgraduate Program in Psychotherapy Research, Department for Psychoanalysis and PsychotherapyMedical University Vienna, Austria

**Keywords:** clinical competence, treatment, triangular space, reverie, psychotherapy

## Introduction

Although it has been shown that psychotherapy is highly effective, there is still a debate on how and why method specific interventions impact on the patient. We propose that psychoanalytic competence is defined by the capability to work with and understand unconscious dynamics by adhering to specific internal attitudes and maintaining certain frames. Due to the elusive nature of the unconscious, establishing an operationalisation of essential competencies is a challenging and intricate task, requiring detailed descriptions of the psychoanalytic process. It is essential to provide an empirically, quantitatively, and qualitatively validatable description. There have been several efforts to operationalize psychoanalytic core competence on a theoretical level as well as empirically. However, these are in many respects too superficial to grasp the full complexity and extent of psychoanalytic concepts of core competence. Although first steps of defining and registering essential core competencies for psychoanalytic practice have been made, we suggest that further developments toward establishing an operationalized empirical research tool are needed.

## Process and conceptual research in psychoanalysis

Advancements in psychoanalytic clinical outcome and process research have produced evidence for the efficiency of psychoanalysis and psychoanalytic psychotherapy (see for instance Gibbons et al., 2008; Shedler, 2010; Gerber et al., 2011). However, there is still a debate on how and why certain interventions change the patient's internal state. In order to contribute to a differentiated understanding of method specific effect factors, various instruments have been implemented. Modern studies most often utilize manual-based treatments, audio, or video recording of sessions, and formal checks on treatment fidelity with instruments such as the PQS (Town et al., 2012). The Psychotherapy Process Q-sort (PQS; Jones, 2000) is designed to assess the process of therapy and quantitatively describes therapy sessions in a manner that captures the complexity of the therapeutic process. With the use of these research-specific procedure instruments, a recent study (Zimmermann et al., 2014), found support for the mediating effect of psychoanalytic techniques, showing that highly favorable long term trajectories of pathological symptoms could be observed. These results support findings from earlier empirical psychotherapy research studies (Ablon and Jones, 1998; Gaston et al., 1998; Hilsenroth et al., 2003). However, although these instruments have made it possible to describe psychoanalytic method and competencies to a certain extent, it is not possible to comprehensively map minute processes between analyst and patient. The PQS is a rather general instrument used to differentiate between different forms of psychotherapy. It is ultimately not able to document complex and multifaceted concepts described in psychoanalytic theory, especially those regarding psychoanalytic core competence.

Therefore, conceptual research studies have aimed at comprising psychoanalytic core competence in a more specific and detailed manner. In conceptual research studies, psychoanalytical concepts are discussed and defined. The aim is to illustrate the power and usefulness of concepts in understanding and structuring clinical material and processes in therapy (Leuzinger-Bohleber and Fischmann, 2006). In searching for a comprehensive definition for the concept of core competencies for psychoanalysts, the aim is to integrate or develop new concepts in order to contribute to a more precise and transparent understanding of the analyst role in the psychoanalytic situation. An implementation and operationalisation of complex psychoanalytic concepts could be accomplished by several steps: First, a description and delimitation regarding general psychotherapeutic competencies is needed. Secondly, an expert based comprehensive description of core competencies has to be obtained based on current theoretical formulations and clinical practice. Here the focus lies in re-evaluating existing definitions of psychoanalytic core competence and finding a consensus on possible advancements in the concepts. The aim is to find an in depth definition that does justice to the complexity and ambiguity the task of working with unconscious processes. Lastly, this description has to be operationalized into an empirically implementable instrument that allows for the analysis of clinical data using the developed categories. Such an instrument would have many beneficial repercussions. It could enable empirical documentation of the way core competencies are used in clinical practice, make it possible to investigate how the dynamics in therapy are affected by the therapists use of these competencies and in what manner they effect the outcome of the treatment.

The first two steps have been conducted on a general level for psychotherapy by the European Association of Psychotherapy (EAP) (2013), and on a psychoanalysis-specific level by the British psychological society's center for outcomes research and effectiveness (CORE) (Lemma et al., 2008). Their report outlines an evidence-based method for identifying competencies, and presents a competence model for psychoanalytic/ psychodynamic therapy. It differentiates generic psychotherapeutic competencies and psychoanalytic ones. Basic psychoanalytic competencies refer to abilities as knowledge of basic principles of analytic approaches, to engage the client in analytic therapy, to work with unconscious communication, to facilitate the exploration of the unconscious dynamics influencing relationships, to be able to work with both the client's internal and external reality. Specific competencies refer to the ability to make interpretations, to work in transference and countertransference and to recognize and work with defenses. This list of competencies was comprised by Expert Reference Group (ERG). In the same manner, the Working Party on Education (WPE) working group on “psychoanalytic competence to practice” discussed and established several essential competence criteria based on modern psychoanalytic theory (Junkers et al., 2008). Tuckett (2005) suggests a possible conceptual framework for describing psychoanalytic core competence further in depth. He focuses on the relevance of a specific mental state and conceptualizes it as comprising three specific capacities or frames.

In the following, these frames are discussed and connected to theoretical concepts in order to illustrate the process of transferring theory to clinical practice and clinical practice back to conceptual development.

## The participant observational, conceptual and interventional frame

The first frame focuses on the manner in which the therapist deals with the patient and what he or she is able to sense in the interaction. Here a central defining aspect is the stance that the therapist takes when listening and the way he or she is capable of understanding the patients as well as their own affects and emotions. Faimberg (1992) argues that “the analyst's overall psychical activity, placed in the service of listening to what the patient says or cannot say during the session” constitutes the “countertransference position.” Tuckett proposes that this capacity lies at the core of maintaining an appropriate participant-observational frame, namely to “countertransferentially “bear” transference” (Tuckett, 2005). The ability to tolerate and to contain the position toward which the analyst is urged by the patient's unconscious transference makes it possible for both to think and wonder about what is happening instead of reacting to it. Current concepts of containment and reverie conceptualize these unconscious dynamics. In these concepts forceful unconscious projective and distorting dynamics in the patient's mind are highlighted and consequently the need for the therapist's capacity for “reverie” (1962). As the patient's destructive phantasies or beta-elements are projected into the therapist, he, or she needs to transform them into alpha elements, a more tolerable affect through his own mental capacity to endure these sensations. If this process of containment goes well, the patient can reintegrate the transformed sensations into his mind. This detailed conceptualization of the curative impact of unconscious communication between patient and therapist emphasizes the importance of the therapist's capacity to understand what happens in the patient's mind. As a result of this process the patient can not only take in a modified version of himself and his emotions but also the therapist in his containing function. Once the patient has integrated the transformed formerly raw and intolerable sensations together with the function of the analyst, they can use them as building blocks for affective development and intellectual growth. Busch also highlights the psychoanalytic quality of thinking essential to effecting change in the patients mind: “Psychoanalytic consciousness is not a static form, but a highly variegated gradation, beginning with an inkling, a dim awareness, that there is a lot going on in one's mind” (Busch and Joseph, 2004). He argues that “processual” knowledge conveys a capacity to think about one's mental activity and to reflect on how internal processes develop (Busch, 2009). This form of knowing derives from an internalization of the psychoanalyst and his mind and ultimately constitutes self-analytic capacity and enables internal development, the “psychoanalytic mind.” It is therefore central to allow for an interweaving of thoughts between the patient and the therapist, an investigation of what happens “here and now” in the session and in the transference and countertransference dynamics. This psychoanalytic mind has been termed the “third” or “analytic” position, which the analyst has to be able to assume in order to create an analytic setting (Bion, 1962). Britton conceptualized the process of creating space for thinking and internal growth described above as the formation of “triangular space” (Britton, 2004). In this transference situation, it is the task of the analyst to accept and realize the patient's projections and to simultaneously conceptualize what is happening by making use of their countertransference feelings. Through this containing process, triangular space can be re-established, as the analyst has made a connection between himself in the outside world and himself as the object of the patient (Schoenhals, 1996).

The competencies discussed above illustrate the internal mindset that is required for working psychoanalytically. The other two frames show the subsequent work of conceptualization and interpretation. Tuckett (2005) highlights that in the conceptual frame, it is crucial to “identify the transference (and countertransference), to conceptualize the development of an analytic process, and to be able to do so in a way that has a “ring of truth” and is not overly intellectual. Essential here is the therapist's capacity to work with the latent meaning of the material in the session and their ability to “conceptualize it theoretically in terms of a psychoanalytic model as required by the specific frame of reference” (Tuckett, 2005). This quest for inner authenticity or truth Bion (1965, 1970) conceptualized in terms of the evolving emotional truth of the psychoanalytic session in the analyst and patient. He argues that psychoanalytic treatment “presupposes that the welfare of the patient demands a constant supply of truth” and “that discovery of the truth about himself is a precondition of an ability to learn the truth, or at least to seek it in his relationship with himself and others. This approach highlights the capacity of the therapist to be capable to conceptualize the patient's unconscious in form of a theory but simultaneously to face the analytic situation without imposing preconceived ideas onto the material. It also means being able to accept and to bear that there is a limitation to what can be known.

Lastly, the interventional frame describes the analyst's capacity to translate this emotional correspondence described above. Interpretations, which “further the kind of psychoanalytic process considered to be transformational within the school in which he is being trained,” are characterized by the right emotional level not being over- intellectualized and showing consistency with what else the analyst is doing (Tuckett, 2005). Such specific interventions are built on the analyst's theory of psychic change or “transformational knowledge” (Körner, 2008). This knowledge derives from a set of concepts that can link the material of the session to a generalizable theory of personality functioning (Meissner, 1984). In empirical research, first steps have been made to document these conceptualizations of interpretation. Hoglend et al. (2008) for instance, isolated “transference interpretations” as an essential core competence and investigated its impact on outcome of psychoanalytic therapies. They were able to show, that especially with patients with weaker object relationship structures, treatment with transference interpretations yielded more favorable results than those using no transference interpretations.

## Conclusion

This paper presents a specific method to conceptualize psychoanalytic core competence, namely the working group based integration and further development of existing kleinian and bionian concepts and theories into a comprehensive description. In addition to the list developed by CORE, Tuckett's approach of focusing on the relevance of a specific psychoanalytic mental state adds an important dynamic dimension. In complementing existing psychoanalytic activity measurement instruments, this approach illustrates the complexity and the challenges of working with, understanding and bearing unconscious processes. Further, similar conceptualizations comprising other psychoanalytic approaches are necessary to create a comprehensive instrument. Developments in empirical research operationalizing these concepts in form of a research tool and implementing them in psychotherapy research studies could contribute to a comprehensive understanding of detailed unconscious processes in the psychoanalytic setting. This could not only contribute to a better empirical documentation of the analytic situation but could moreover contribute to discussions on psychoanalytic training and education as well as further interdisciplinary discussion. As the research presented above has conducted essential conceptual research steps in identifying and defining psychoanalytic core competence, in a subsequent step, these lists could be operationalized in form of an instrument similar to the PQS with the novelty of being tailored to describing the processes discussed above.

### Conflict of interest statement

The authors declare that the research was conducted in the absence of any commercial or financial relationships that could be construed as a potential conflict of interest.
